# Supplementary material for: Towards Optimized Photoluminescent Copper(I) Phenanthroline-Functionalized Complexes: Control of the Photophysics by Symmetry-Breaking and Spin–Orbit Coupling
Source: Materials (Basel). 2022 Jul 28;15(15):5222. doi: 10.3390/ma15155222 (PMC9369739; doi:10.3390/ma15155222)
Supplement: Supplementary file 1 [file materials-15-05222-s001.zip › Supplementary information-Final.pdf]

Supplementary Information

Towards optimized photoluminescent Copper(I) phenanthroline-functionalized complexes: Control of the photophysics by symmetry breaking and spin-orbit coupling

Christophe Gourlaouen [1,*] and Chantal Daniel[1]

Affiliation 1: Laboratoire de Chimie Quantique Institut de Chimie UMR 7177 CNRS-Université de Strasbourg,
4, Rue Blaise Pascal CS 90032, F-67081 Strasbourg Cedex, France.
* Co: gourlaouen@unistra.fr

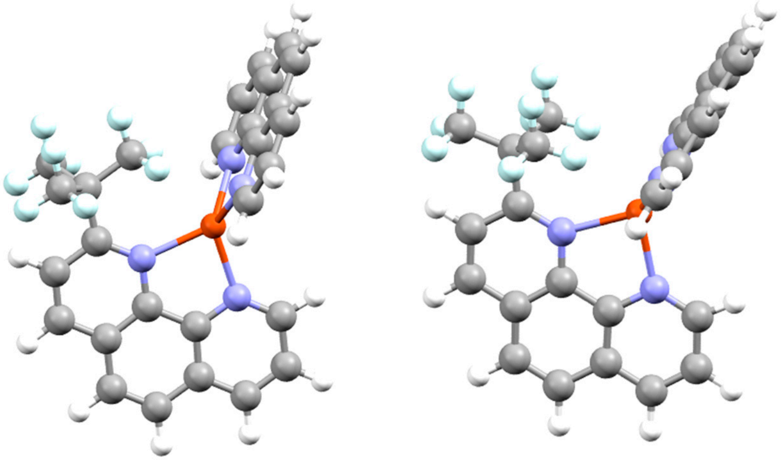

Figure S1: In (left) and out (right) orientations of a tBu group on a simplified complex holding only one tBu.

Structure	DEVVIJ[1]	DEVVIJ	BUFSOM[2]	BUZRIZ[3]	GATYUX[4]	MAHBOP[5]	NADGAA[6]	ELEZUS[7]	IBISOC[8]	1_H
Cu-N	2.047	2.035	1.996	2.046	2.026	2.009	2.033	2.036	2.082	2.068
Cu-N	2.059	2.054	1.996	2.040	2.058	2.069	2.061	2.015	2.048	2.068
Cu-N	2.047	2.035	1.996	2.027	2.067	2.081	2.034	2.015	2.055	2.068
Cu-N	2.059	2.054	1.996	2.055	2.009	1.998	2.068	2.036	2.079	2.068
$Cu-X_1$	3.982	3.972	3.924	4.000	3.966	3.947	3.975	3.943	4.003	4.002
$Cu-X_2$	3.982	3.972	3.924	3.990	3.960	3.959	3.969	3.943	4.002	4.002
X_1-Cu-X_2	175.8	174.6	180.0	179.0	163.6	144.8	178.2	171.1	178.7	180.0
$C_1-X_1-X_2-C_2$	58.1	71.0	33.9	87.6	90.0	67.2	47.6	58.9	42.8	90.0

Table S1: Experimental (from CSD reference) and computed (GAUSSIAN) geometrical parameters for complex **1_H.** Distances are in Angstroms, angles and dihedral angles in degrees.

Structure	DMPRCU[9]	DMPRCU	DMPNCU[10]	ABAFAN[11]	HEVPAA[12]	IPICUF[13]	NILHUL[14]	1_{Me}
Cu-N	2.061	2.058	2.008	2.012	1.932	2.013	2.022	2.060
Cu-N	2.043	2.084	2.140	2.032	2.011	2.022	2.025	2.060
Cu-N	2.043	2.058	2.062	2.020	2.026	1.997	2.022	2.060
Cu-N	2.061	2.084	2.064	2.027	2.011	2.045	2.025	2.060
Cu-X_1	3.980	3.995	3.995	3.952	3.921	3.942	3.945	3.994
Cu-X_2	3.980	3.995	3.960	3.948	3.939	3.940	3.945	3.994
X_1-Cu-X_2	171.4	171.8	151.6	170.5	171.6	165.0	176.9	180.0
C_1-X_1-X_2-C_2	67.6	68.3	78.3	74.7	87.0	89.9	81.0	90.0

Table S2: Experimental (from CSD reference) and computed (GAUSSIAN) geometrical parameters for complex $\mathbf{1_{Me}}$**.** Distances are in Angstroms, angles and dihedral angles in degrees.

Structure	α	β	γ	δ	ζ	ε	2	3
Cu-N	2.075	2.141	2.113	2.129	2.144	2.144	2.155	2.035
Cu-N	2.075	2.141	2.133	2.161	2.144	2.146	2.155	2.290
Cu-N	2.075	2.142	2.120	2.129	2.147	2.181	2.169	2.114
Cu-N	2.075	2.142	2.121	2.161	2.147	2.114	2.169	2.114
Cu-X_1	3.931	4.032	3.998	4.033	4.037	4.036	4.081	4.095
Cu-X_2	3.931	4.035	4.005	4.033	4.041	4.042	4.083	4.028
X_1-Cu-X_2	180.0	180.0	172.4	172.4	180.0	178.2	180.0	176.4
C_1-X_1-X_2-C_2	90.0	84.7	89.1	88.8	90.0	88.4	90.0	90.0

Table S3: Computed (GAUSSIAN) geometrical parameters for complexes $\mathbf{1_{tBu}}$**, 2** and **3** in the ground state. Distances are in Angstroms, angles and dihedral angles in degrees.

Structure	EDOFEJ[15]				
	δ	δ	δ	ζ	ε
Cu-N	2.103	2.076	2.081	2.105	2.072
Cu-N	2.120	2.139	2.105	2.108	2.145
Cu-N	2.096	2.096	2.092	2.104	2.148
Cu-N	2.129	2.115	2.114	2.111	2.079
Cu-X_1	3.992	3.984	3.976	4.005	4.002
Cu-X_2	3.999	3.997	3.980	3.990	4.000
X_1-Cu-X_2	174.6	170.2	176.6	177.0	176.0
C_1-X_1-X_2-C_2	81.2	89.6	88.5	86.6	85.7

Table S4: Experimental geometrical parameters for complex $\mathbf{1_{tBu}}$**.** Distances are in Angstroms, angles and dihedral angles in degrees.

Structure	1_H	1_H (TS1)	1_{Me}	1_{Me} (TS1)	1_{Me} (TS2)	1_{tBu}	1_{tBu} (TS2)	2	3
Cu-N	1.993	1.937	1.969	1.937	1.994	1.987	2.034	2.000	1.975
Cu-N	1.993	1.944	1.969	1.939	1.994	2.000	2.073	2.000	1.975
Cu-N	1.993	2.118	2.027	2.109	1.994	2.129	2.034	2.219	2.093
Cu-N	1.993	2.118	2.027	2.109	1.994	2.231	2.073	2.219	2.345
Cu-X_1	3.934	3.881	3.906	3.879	3.927	3.897	3.948	3.914	3.900
Cu-X_2	3.934	4.063	3.955	4.050	3.927	4.084	3.948	4.164	4.167
X_1-Cu-X_2	180.0	161.1	180.0	179.1	180.0	168.8	176.1	180.0	176.7
C_1-X_1-X_2-C_2	41.5	90.0	65.0	90.0	62.8	86.3	82.9	90.0	90.0
ΔG	0.0	12.9	0.0	4.4	-0.4	0.0	2.4		

Table S5: Computed (GAUSSIAN) geometrical parameters for complexes in the triplet state. The values are given for conformer δ for $\mathbf{1}_{tBu}$. Distances are in Angstroms, angles and dihedral angles in degrees and energies in kcal mol^{-1}.

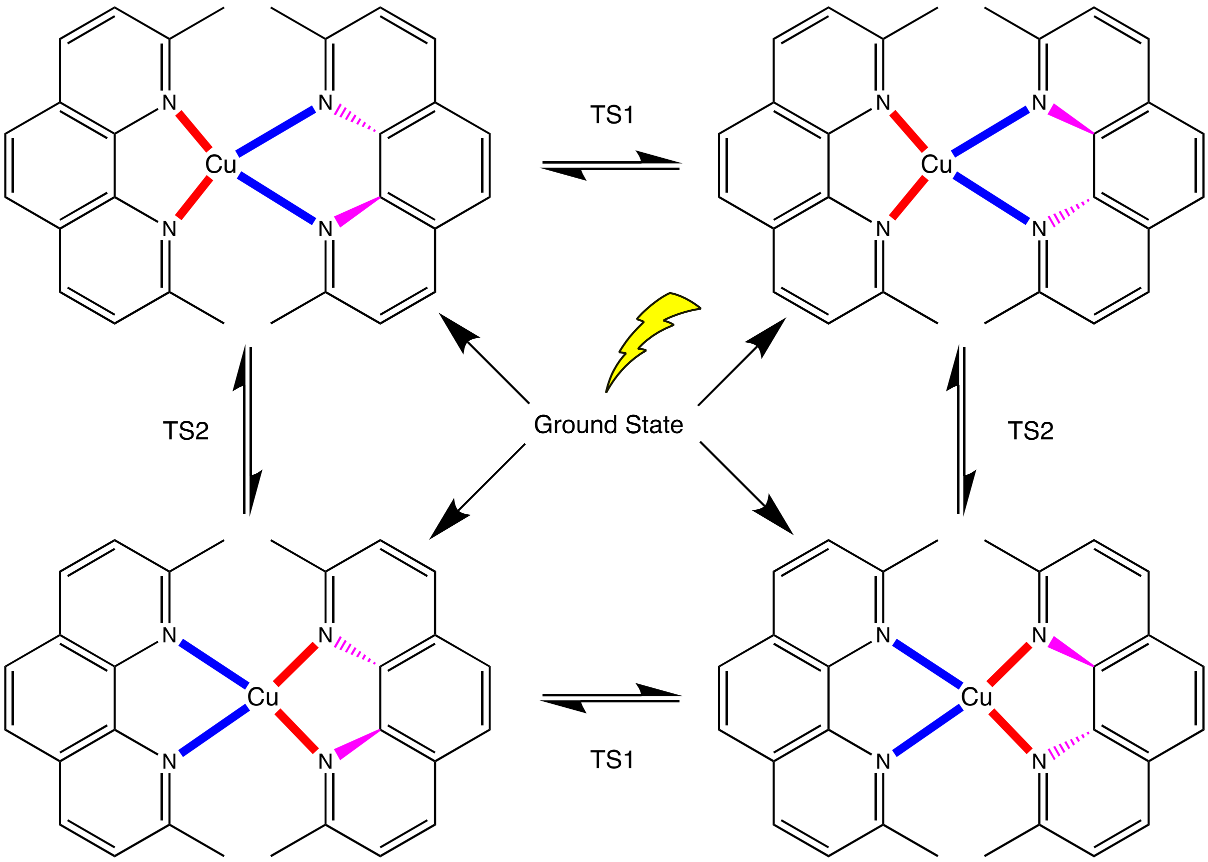

Scheme S1: Representation of the four possible Triplet minima after irradiation of $\mathbf{1}_{Me}$ complex. In bold blue are the long Cu-N distance, in bold red the short Cu-N distance and in purple the indication of how the phen ligands is out of plane.

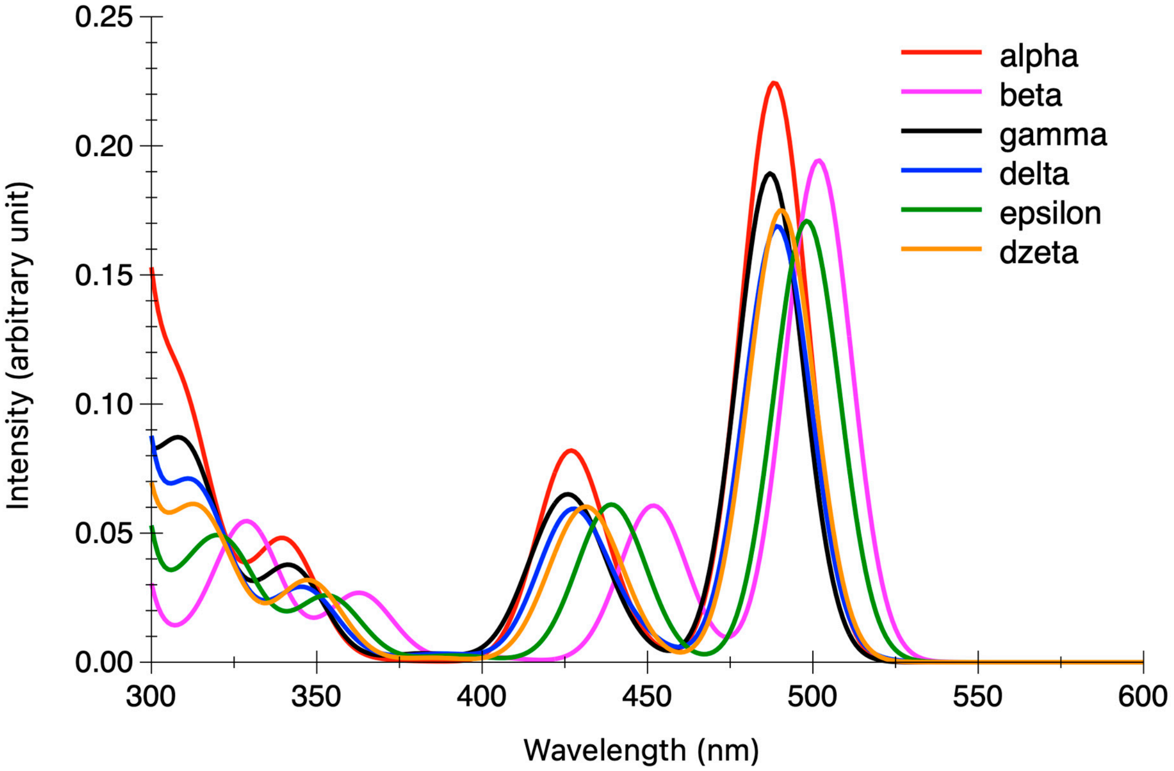

Figure S2: ADF computed Absorption spectra for the different conformers of **1_{tBu}.**

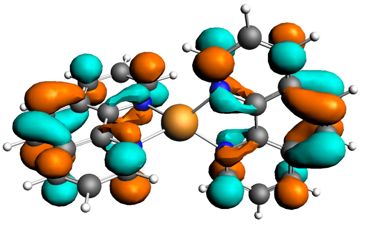	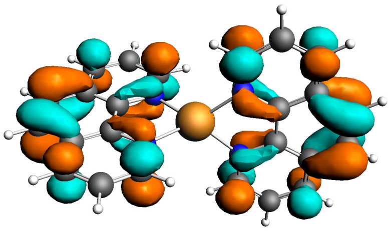
LUMO+2, -2.281	LUMO+3, -2.279
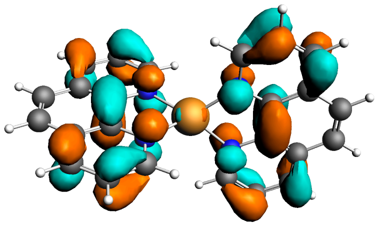	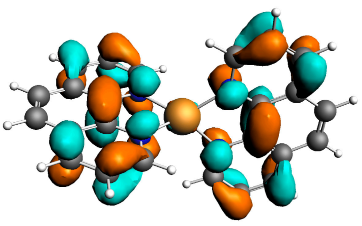
LUMO, -2.412	LUMO+1, -2.412
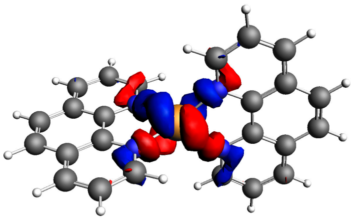	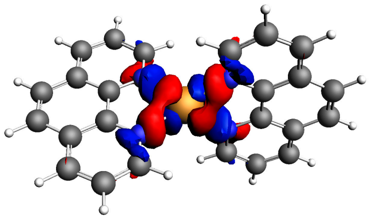
HOMO-1, -5.754	HOMO, -5.754

Figure S3: Frontier orbitals of **1_H** and energies in eV.

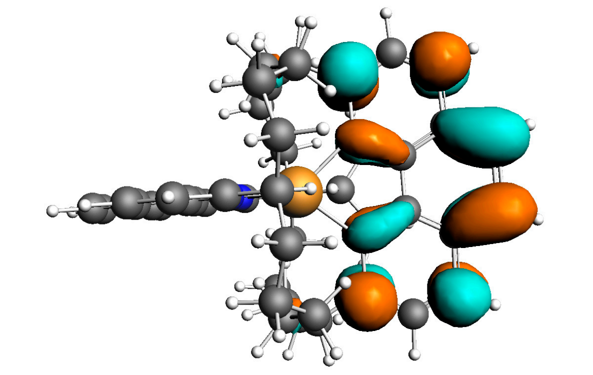	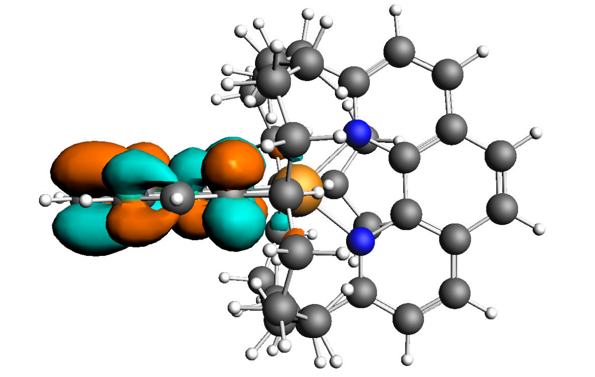
LUMO+2, -2.227	LUMO+3, -2.133
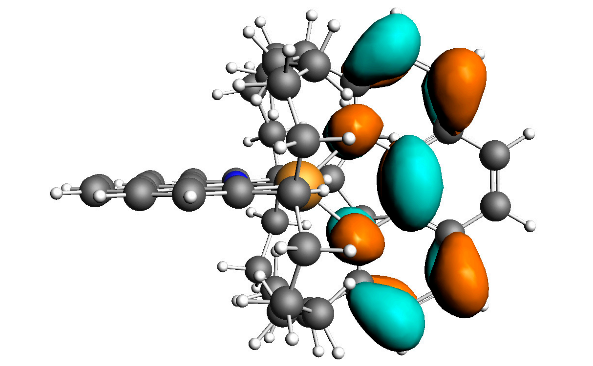	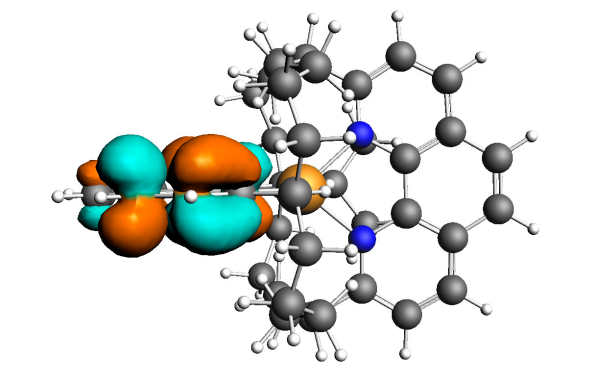
LUMO, -2.476	LUMO+1, -2.359
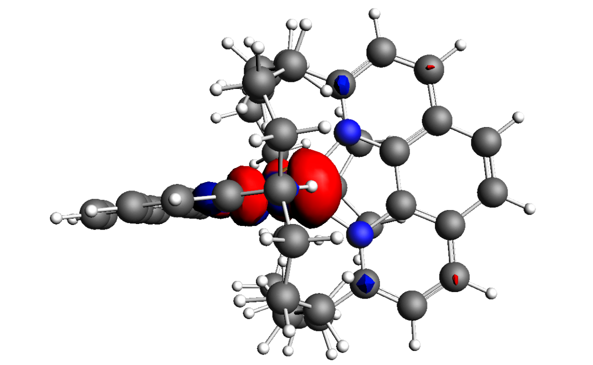	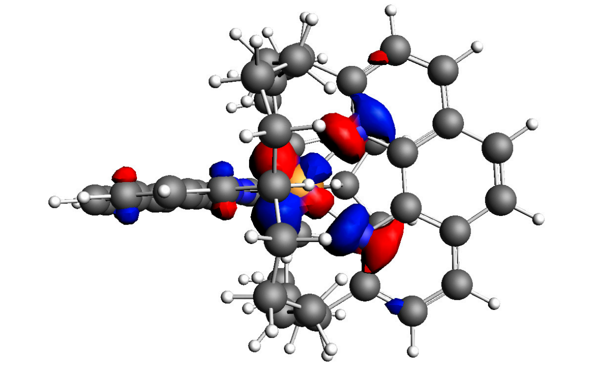
HOMO-1, -5.899	HOMO, -5.825

Figure S4: Frontier orbitals of **3** and energies in eV.

	S0 (D2d)	S1	T1	T2
Cu-N	2.062	2.004	1.991	2.028
	2.062	2.004	1.991	2.028
	2.062	2.064	2.035	2.028
	2.062	2.064	2.035	2.028
Cu-X_1	3.989	3.933	3.931	3.957
Cu-X_2	3.989	3.993	3.962	3.957

X_1-Cu-X_2	180.0	179.9	179.9	180.0
C_1-X_1-X_2-C_2	90.0	39.9	39.9	40.4

Table S6: Selected nuclear coordinates at the optimized ground state and low-lying excited states for **1_H** computed with ADF. Distances are in Angstroms, angles and dihedral angles in degrees.

	S0 (D2d)	S1	T1	T2
Cu-N	2.056	1.985	1.990	2.019
	2.056	1.985	1.990	2.019
	2.056	2.102	2.047	2.020
	2.056	2.102	2.047	2.020
Cu-X_1	3.980	3.900	3.922	3.940
Cu-X_2	3.980	4.027	3.965	3.940
X_1-Cu-X_2	180.0	180.0	180.0	180.0
C_1-X_1-X_2-C_2	90.0	68.2	65.0	68.1

Table S7: Selected nuclear coordinates at the optimized ground state and low-lying excited states for **1_{Me}** computed with ADF. Distances are in Angstroms, angles and dihedral angles in degrees.

	S0 (C2)	S1	T1	T2
Cu-N	2.118	2.005	1.996	2.035
	2.153	2.022	2.021	2.158
	2.118	2.171	2.168	2.032
	2.153	2.306	2.298	2.161
Cu-X_1	4.017	3.898	3.900	3.987
Cu-X_2	4.017	4.142	4.134	3.988
X_1-Cu-X_2	172.5	167.9	167.6	166.6
C_1-X_1-X_2-C_2	89.1	88.1	86.0	83.0

Table S8: Selected nuclear coordinates at the optimized ground state and low-lying excited states for **1_{tBu}** for experimental δ structure computed with ADF. Distances are in Angstroms, angles and dihedral angles in degrees.

**Structure**	**1_H**	**1_{Me}**	**1_{tBu} (α)**	**1_{tBu} (β)**	**1_{tBu} (γ)**	**1_{tBu} (δ)**	**1_{tBu} (ε)**	**1_{tBu} (ζ)**	**2**	**3**
Cu-N	2.062	2.056	2.064	2.129	2.107	2.118	2.134	2.136	2.156	2.102
Cu-N	2.062	2.056	2.074	2.135	2.115	2.153	2.137	2.141	2.156	2.116
Cu-N	2.062	2.056	2.064	2.129	2.107	2.118	2.182	2.132	2.140	2.020
Cu-N	2.062	2.056	2.074	2.135	2.118	2.153	2.102	2.137	2.140	2.299
Cu-X_1	3.989	3.980	3.919	4.019	3.981	4.017	4.023	4.025	4.061	4.018
Cu-X_2	3.989	3.980	3.919	4.019	3.988	4.017	4.030	4.021	4.059	4.083
X_1-Cu-X_2	180.0	180.0	179.7	179.7	173.1	172.5	179.4	179.9	180.0	174.4
C_1-X_1-X_2-C_2	90.0	90.0	89.6	89.3	89.5	89.1	89.6	89.3	90.0	89.7

Table S9: Computed ground state (ADF) geometrical parameters. Distances are in Angstroms, angles and dihedral angles in degrees.

State	Conformer	α	β	γ	γ^1	δ	ε	ε^1	ζ	ζ^1
S1	Cu-N	1.980	2.022	1.996	2.189	2.005	2.016	2.215	2.018	2.223
	Cu-N	1.980	2.022	2.000	2.209	2.022	2.018	2.257	2.018	2.223
	Cu-N	2.139	2.239	2.145	2.002	2.171	2.229	2.017	2.222	2.01
	Cu-N	2.139	2.239	2.281	2.014	2.306	2.255	2.018	2.222	2.01
	Cu-X_1	3.839	3.908	3.873	4.082	3.898	3.901	4.139	3.908	4.126
	Cu-X_2	3.998	4.144	4.105	3.887	4.142	4.149	3.906	4.121	3.892
	X_1-Cu-X_2	180.0	180.0	169.4	170.6	167.9	176.3	179.1	180.0	180.0
	C_1-X_1-X_2-C_2	89.0	88.8	88.7	89.4	88.1	90.0	89.7	89.9	89.8
T1	Cu-N	1.985	2.015	1.989	2.165	1.996	2.008	2.210	2.010	2.220
	Cu-N	1.985	2.015	1.993	2.225	2.021	2.009	2.259	2.010	2.220
	Cu-N	2.108	2.237	2.154	2.012	2.168	2.221	2.008	2.220	2.003
	Cu-N	2.108	2.237	2.256	1.990	2.298	2.263	2.011	2.220	2.003
	Cu-X_1	3.853	3.908	3.873	4.077	3.900	3.899	4.136	3.907	4.122
	Cu-X_2	3.963	4.140	4.097	3.888	4.134	4.142	3.904	4.117	3.891
	X_1-Cu-X_2	180.0	180.0	170.4	170.1	167.6	175.8	179.2	180.0	180.0
	C_1-X_1-X_2-C_2	87.1	89.0	89.4	88.4	86.0	89.1	89.6	89.9	89.9

Table S10: Selected nuclear coordinates at the low-lying excited states for $\mathbf{1}_{\mathbf{tBu}}$ computed with ADF. Distances are in Angstroms, and angles in degrees.

State	Conformer	α	β	γ	γ^1	δ	ε	ε^1	ζ	ζ^1
	GS Symmetry	D_{2d}	D_{2d}	C_1	C_1	C_2	C_1	C_1	C_{2v}	C_{2v}
S1	S1 Symmetry	C_2	C_2	C_1	C_1	C_1	C_1	C_1	C_2	C_2
	E_{def}	0.542	0.497	0.279	0.287	0.286	0.376	0.374	0.249	0.244
	E_{em}	1.944	1.779	1.870	1.897	1.813	1.801	1.826	1.884	1.863
	E_{stab}	2.486	2.275	2.149	2.184	2.099	2.177	2.200	2.133	2.106
	ΔE	0.387	0.176	0.050	0.085	0.000	0.078	0.101	0.033	0.007
	λ_{em}	638	697	663	653	684	688	679	658	666
	f_{osc}	$1.06\ 10^{-5}$	$1.57\ 10^{-6}$	$1.53\ 10^{-5}$	$1.16\ 10^{-4}$	$2.54\ 10^{-4}$	$2.53\ 10^{-5}$	$2.20\ 10^{-7}$	$1.59\ 10^{-8}$	$1.07\ 10^{-7}$
T1	T1 Symmetry	C_2	C_2	C_1	C_1	C_1	C_1	C_1	C_2	C_2
	E_{def}	0.585	0.513	0.295	0.314	0.341	0.397	0.397	0.270	0.263
	E_{em}	1.745	1.633	1.715	1.731	1.656	1.648	1.671	1.726	1.707
	E_{stab}	2.331	2.146	2.011	2.044	1.997	2.046	2.068	1.997	1.970
	ΔE	0.360	0.175	0.040	0.074	0.027	0.075	0.098	0.026	0.000
	λ_{em}	710	759	723	716	749	752	742	718	726
	ΔE_{ST}	0.188	0.133	0.144	0.147	0.143	0.138	0.136	0.140	0.141
	SOC S1-T1	73.0	6.0	3.6	19.7	20.1	8.3	0.64	0.0	1.0
		$3.04\ 10^{-6}$	$2.10\ 10^{-9}$	$7.05\ 10^{-7}$	$7.42\ 10^{-6}$	$6.85\ 10^{-6}$	$4.25\ 10^{-6}$	$3.93\ 10^{-6}$	$1.09\ 10^{-13}$	$2.92\ 10^{-10}$
	f_{osc}	$8.97\ 10^{-6}$	$6.86\ 10^{-6}$	$4.69\ 10^{-5}$	$1.43\ 10^{-5}$	$2.77\ 10^{-5}$	$1.42\ 10^{-5}$	$2.04\ 10^{-4}$	$6.25\ 10^{-6}$	$7.43\ 10^{-6}$
		$3.04\ 10^{-4}$	$3.01\ 10^{-4}$	$2.81\ 10^{-4}$	$2.52\ 10^{-4}$	$1.77\ 10^{-04}$	$2.46\ 10^{-4}$	$3.98\ 10^{-5}$	$2.88\ 10^{-4}$	$3.77\ 10^{-4}$

Table S11: Emission data computed with ADF for S1 and T1 states of $\mathbf{1}_{\mathbf{tBu}}$. E_{def}, E_{em}, E_{stab}, ΔE and ΔE_{ST} are in eV, λ_{em} is in nm, SOC is in cm^{-1}. 1) Values for the 2nd minima due to oscillation of the exciton for γ, ε and ζ. Data are not given for α, β and δ because the second minima is strictly identical to the first one due to symmetry.

	S1	S2	S3	S4	T1	T2	T3	T4
**T1**	0.0	0.0	302.0	6.8	0.0	0.0	0.0	381.0
**T2**	269.0	0.0	0.0	21.6	0.0	0.0	421.1	0.0
**T3**	0.0	0.0	0.0	18.7	0.0	421.1	0.0	0.0
**T4**	0.0	295.8	0.0	10.3	381.1	0.0	0.0	0.0

Table S12: Spin-orbit coupling (in cm^{-1}) between the low-lying excited states in $\mathbf{1}_{Me}$ computed with ADF.

	Structure	2	2	3	3
S1	Cu-N	2.017	2.271	1.989	2.200
	Cu-N	2.017	2.271	1.996	2.209
	Cu-N	2.254	2.017	2.122	1.980
	Cu-N	2.254	2.017	2.364	1.999
	Cu-X_1	3.910	4.193	3.899	4.132
	Cu-X_2	4.194	3.932	4.182	3.906
	X_1-Cu-X_2	180.0	180.0	174.9	176.5
	C_1-X_1-X_2-C_2	90.0	90.0	89.3	89.7
T1	Cu-N	2.010	2.269	1.978	2.205
	Cu-N	2.010	2.269	1.995	2.193
	Cu-N	2.250	2.011	2.120	1.973
	Cu-N	2.250	2.011	2.354	1.988
	Cu-X_1	3.908	4.192	3.899	4.125
	Cu-X_2	4.189	3.933	4.177	3.903
	X_1-Cu-X_2	180.0	180.0	175.1	176.5
	C_1-X_1-X_2-C_2	90.0	89.7	88.9	89.8

Table S13: Computed S1 and T1 (ADF) geometrical parameters. Distances are in Angstroms, angles and dihedral angles in degrees.

---

[1] Wang, R.Z.; Xu, J.Q.; Yang, G.Y.; Bu, W.M.; Xing, Y.H.; Li, D.M.; Liu, S.Q.; Ye, L.; Fan, Y.G. Structure of [Cu(phen)2]2[{Cu(phen)}2Mo8o26].H2O. *Pol. J. Chem.* **1999**, *73*, 1909.

[2] Avdeeva, V.V.; Dziova, A.E.; Polyakova, I.N.; Malinina, E.A.; Goeva, L.V.; Kuznetsov, N.T. Copper(I), copper(II), and heterovalent copper(I,II) complexes with 1,10-phenanthroline and the closo-decaborate anion. *Inorg. Chim. Acta* **2015**, 430, 74–81

[3] Rheingold, A.L. (Department of Chemistry, University of California, La Jolla, CA, USA); Maatta, E. (Department of Chemistry, Kansas State University, Manhattan, KS, USA) CSD Communication, 2015.

[4] Huang, Z.; Hartwig, J.F. Copper(I) Enolate complexes in α-Arylation Reactions: Synthesis, Reactivity and Mechanism. *Angew. Chem. Int. Ed.* **2012**, *51*, 1028–1032.

[5] Lin, X.; Hou, C.; Li, H.; Weng, Z. Decarboxylative Trifluoromethylating Reagent [Cu(O2CCF3)(phen)] and Difluorocarbene Precursor [Cu(phen)2][O2CCF2Cl]. *Chem. Eur. J.* **2016**, *22*, 2075-2084.

[6] Hillhouse, G. (Searle Chemistry Laboratory, Department of Chemistry, The University of Chicago, Chicago, IL, USA); Rheingold, A.L. (Department of Chemistry, University of Delaware, Newark, DE, USA); Allen, M.B. CSD Communication, 1996

[7] Huang, Y.; Ajitha, M.J.; Huang, K.-W.; Zhang, Z.; Weng, Z. A class of effective decarboxylative perfluoroalkylating reagents: [(phen)2Cu](O2CRF) *Dalton Trans.* **2016**, *45*, 8468-8474

[8] Yu, J.-H.; Lu, Z.-L.; Xu, J.-Q.; Bie, H.-Y.; Lu, J.; Zhang, X. Syntheses, characterization and optical properties of some copper(i) halides with 1,10-phenanthroline ligand, *New J. Chem.* **2004**, *28*, 940-945

[9] Hamalainen, R. (Department of Inorganic Chemistry, University of Helsinki, Helsinki, Finland); Ahlgren, M. (Department of Inorganic Chemistry, University of Helsinki, Helsinki, Finland); Turpeinen, U. (Department of Inorganic Chemistry, University of Helsinki, Helsinki, Finland); Raikas, T (Department of Inorganic Chemistry, University of Helsinki, Helsinki, Finland). Crystal Structure Communications. 1979.

[10] Dessy, G. (Laboratorio di Teoria e Struttura Elettronica e Comportamento Spettrochimico dei Composti di Coordinazione del CNR, Monterotondo Stazione, Italy); Fares, V. (Laboratorio di Teoria e Struttura Elettronica e Comportamento Spettrochimico dei Composti di Coordinazione del CNR, Monterotondo Stazione, Italy). Crystal Structure Communications, 1979.

[11] Royappa, A.T.; Royappa, A.D.; Moral, R.F.; Rheingold, A.L.; Papoular, R.J.; Blum, D.M.; Duong, T.Q.; Stepherson, J.R.; Vu, O.D.; Chen, B.; Suchomel, M.R.; et al. Copper(I) oxalate complexes: Synthesis, structures and surprises *Polyhedron*, **2016**, *119*, 563-574.

[12] Zheng, S.-L.; Gembicky, M.; Messerschmidt, M.; Dominiak, P.M.; Coppens, P. Effect of the Environment on Molecular Properties: Synthesis, Structure, and Photoluminescence of Cu(I) Bis(2,9-dimethyl-1,10-phenanthroline) Nanoclusters in Eight Different Supramolecular Frameworks. *Inorg. Chem.* **2006**, *45*, 9281-9289

[13] Kovalevsky, A. Y.; Gembicky, M.; Novozhilova, I. V.; Coppens, P. Solid-State Structure Dependence of the Molecular Distortion and Spectroscopic Properties of the Cu(I) Bis(2,9-dimethyl-1,10-phenanthroline) Ion. *Inorg. Chem.* **2003**, *42*, 8794-8802

[14] Blake, A.J.; Hill, S.J.; Hubberstey, P.; Li, W.-S. Copper(I) chelated by 2,9-dimethyl-1,10-phenanthroline and bridged by 4,4′-bipyridine or trans-1,2-bis(pyridin-4-yl)ethene to give discrete dinuclear and polymeric cations. *J. Chem. Soc. Dalton Trans.* **1998**, 909-916

[15] Gandhi, B.A.; Green, O.; Burstyn, J.N. Facile oxidation-based of sterically encumbered four-coordinate bis(2,9-di-*ter*-butyl-1,10-phenanthroline)copper(I) and related three-coordinate copper(I) complexes. *Inorg. Chem.* **2007**, *46*, 3816–3825.
